# The effects of social media addiction on depression and anxiety among university students: The mediating role of family environment

**DOI:** 10.1038/s41598-026-45666-z

**Published:** 2026-03-24

**Authors:** Arif Jameel, Huiji Xu, Wenjing Guo, Shahreen Malik, Noman Sahito

**Affiliations:** 1 School of Business, Shandong Xiehe University, Jinan, 250109 P.R. China; 2 Department of Management Sciences, COMSATS University Islamabad - Lahore Campus, Lahore, Pakistan; 3 Architecture and City Designing Department College of Design and Built Environment, King Fahd University of Petroleum and Minerals Dhahran, Dhahran, Saudi Arabia

**Keywords:** Social media, Depression, Anxiety, Family environment, University students, Health care, Psychology, Psychology

## Abstract

The significant mental health concerns have been highlighted by the rising incidence of social media addiction among university students. This research examined the relationship between social media addiction (SMA) and both depression and anxiety, while exploring the mediating effect of the family environment (FE) among Saudi university students. A cross-sectional survey was conducted between January and February 2025 among 627 students from four Saudi public universities (376 men and 251 women). Using SPSS 25.0 and AMOS 25.0, descriptive, correlational, and mediation analyses were conducted. Significant positive relationships were seen between SMA and anxiety (*r* = 0.41, *p* < 0.001) and depression (*r* = 0.37, *p* < 0.001). Additionally, there was a strong correlation between the family environment and anxiety (*r* = 0.42, *p* < 0.001) and depression (*r* = 0.31, *p* < 0.001). The indirect effects were statistically significant (bootstrapped 95% CI not containing zero), and mediation analysis showed that FE fully mediated the connections between SMA and both anxiety and depression. These results highlight the need for early treatments that address students’ social media-related discomfort while creating a nurturing home setting to mitigate the psychological effects of excessive social media use.

## Introduction

Social media platforms such as Snapchat, Meta, and Instagram are widely used among Saudi university students^1,2^. Concerns have emerged regarding the impact of this usage on students’ mental health, particularly its relationship with depression and anxiety^3,4^. Overuse of social media has sparked public concern about Social Media Addiction (SMA), a behavioral trend that is becoming more widely acknowledged among youngsters globally. However, SMA prevalence rates range from roughly 17 to 27% in China, the US, Japan, and Turkey^5–7^. However, direct comparison is challenging because of variations in study design, sample, and measuring methods. However, taken as a whole, these numbers demonstrate how widespread this problem is. Although SMA is not formally classified as a mental disorder by the American Psychiatric Association (APA), Mazzella, Curry, Belter, Cruz and Jarecki^8^, the World Health Organization’s inclusion of gaming disorder in the ICD-11 Reed, First, Billieux, Cloitre, Briken, Achab, Brewin, King, Kraus and Bryant^9^ has intensified debate over behavioral addictions. A precise, universally accepted definition of SMA has yet to emerge Musetti, Cattivelli, Giacobbi, Zuglian, Ceccarini, Capelli, Pietrabissa and Castelnuovo^10^, but it generally refers to persistent, uncontrollable social media use that interferes with daily life. Most existing studies on social media addiction have been conducted in Western (WEIRD) populations, leaving a limited understanding of how cultural, familial, and societal factors shape this behavior in non-WEIRD settings such as Saudi Arabia. Saudi youth represent a particularly relevant population given the nation’s rapid digitalization and strong family-oriented culture. SMA has been associated with maladaptive behaviors such as aggression Chung, Lee and Lee^11^ and Obeid, Saade, Haddad, Sacre, Khansa, Al Hajj, Kheir and Hallit^12^, impulsivity Obeid, Saade, Haddad, Sacre, Khansa, Al Hajj, Kheir and Hallit^12^, and suicidal ideation Pan and Yeh^13^, as well as emotional problems including depression Olivier, Morin, Tardif-Grenier, Archambault, Dupéré and Hébert^14^, stress Gao, Teng, Wei, Jin, Xiao, Tang, Wu, Yang, Yan and Chen^15^, and anxiety^16^.

The present study used two theoretical frameworks to explain these relationships. First, according to the mood enhancement theory, people who are feeling down might turn to social media to feel better or to get away from their problems. Although this could offer short-term respite, a constant reliance on social media increases susceptibility to addiction and perpetuates dependence. Second, according to the social comparison theory, people assess themselves by contrasting their online lives with those of others. Social media exposure to idealized lives can erode self-worth and increase anxiety and sadness. When combined, these theories offer a thorough framework for comprehending the psychological mechanisms that connect SMA to university students’ mental health outcomes.

**Fig. 1:**
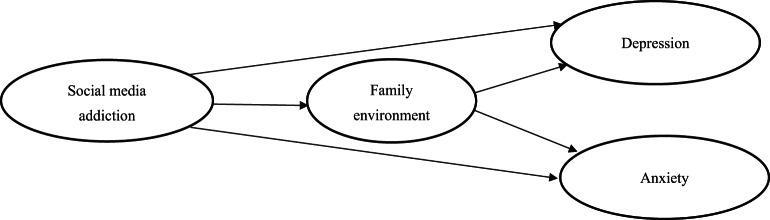
Conceptual Framework of the Study.

A significant portion of psychological research has focused on WEIRD populations, creating replication and generalizability issues^17^. According to Henrich, Heine and Norenzayan^17^, WEIRD populations make up just around 12% of the world’s population; cross-cultural research is essential to expanding theoretical knowledge. Given that SMA is influenced by academic pressure Jun and Choi^18^, cultural variation Sokołowska^19^, and social context Fumero, Marrero, Voltes and Peñate^20^, research among non-WEIRD populations, such as Saudi Arabia, is needed. Accordingly, the present research investigates the associations between social media addiction, depression, and anxiety among Saudi university students and examines the mediating role of the family environment in these relationships. This research extends prior work by providing culturally grounded evidence from a non-WEIRD context, where family and social norms strongly influence youth behavior. The proposed conceptual model is presented in Fig. 1.

## Literature review and hypotheses development

### Social media addiction and depression

Social media addiction has become a pressing public-health concern because of its significant association with depressive symptoms. Excessive and uncontrolled engagement with social platforms has been shown to intensify loneliness, anxiety, and emotional isolation—key risk factors for depression^21–23^. Frequent exposure to idealized online portrayals often triggers social comparisons that distort self-perception and foster dissatisfaction and sadness among young users^24^. According to social comparison theory, individuals evaluate their self-worth relative to others, and these upward comparisons can diminish self-esteem and heighten depressive tendencies^25^. Empirical evidence further supports this link: greater daily time spent on social media predicts stronger depressive symptoms, suggesting a dose–response pattern^26^. Collectively, these findings indicate that social-media addiction exacerbates depressive outcomes by reinforcing maladaptive comparison processes and emotional withdrawal.

#### H1

Social-media addiction is significantly associated with higher levels of depression.

### Social media addiction and anxiety

Beyond depressive symptoms, social-media addiction has also been identified as a major predictor of anxiety among adolescents and young adults. Persistent engagement with social networks often heightens anxiety through mechanisms such as fear of missing out, cyberbullying exposure, and continuous self-evaluation^27–29^. Compulsive use creates a feedback loop in which users struggle to disengage, thereby amplifying anxiety and dependence^30^. Moreover, the curated nature of online content encourages unrealistic comparisons and perceived inadequacy, while simultaneously discouraging effective coping and emotional regulation^31^. Individuals with greater addiction to social media thus become more susceptible to online stressors, leading to chronic anxiousness and restlessness.

#### H2

Social-media addiction is significantly associated with higher levels of anxiety.

### Mediating role of family environment

Recent research highlights the mediating role of the family environment in the relationship between social-media addiction and mental-health disorders such as anxiety and depression. Grounded in Social Comparison Theory and Mood Enhancement Theory, excessive social-media engagement can distort individuals’ self-perceptions and emotional regulation, making them more vulnerable to negative psychological outcomes. In line with Social Comparison Theory, individuals’ reactions to online comparisons and their ability to manage resulting emotions are strongly shaped by the quality of their family environment. Excessive social media usage can lead to feelings of inadequacy, anxiety, and sadness through constant social comparison and exposure to cyberbullying^32,33^. However, the degree of harm varies greatly depending on family dynamics. A supportive family environment characterized by open communication, emotional warmth, and parental involvement can buffer these effects, whereas dysfunctional family relationships intensify them^34–36^. Empirical findings further show that parental monitoring and guidance have been shown to significantly reduce the likelihood of anxiety and depression related to online activity^37,38^. Family-based interventions, such as promoting offline social interaction and digital-use boundaries, also mitigate these adverse outcomes^39,40^.

Therefore, based on these theories, the family environment functions as a psychosocial mechanism that translates the effects of social-media addiction into mental-health outcomes by shaping emotional responses and coping behaviors. Together, this evidence suggests that a positive family environment mediates the relationship between social-media addiction and mental-health outcomes, highlighting the value of family support as a protective mechanism.

H3: Family environment mediates the relationship between social media addiction and depression.

H4: Family environment mediates the relationship between social media addiction and anxiety.

### Conceptual framework

Building on the literature and theoretical foundations, Figure 1 illustrates the proposed conceptual framework. The model hypothesizes that social media addiction is positively associated with depression and anxiety, while the family environment plays a mediating role in these relationships.

## Methodology

### Research procedure

A multi-stage stratified random sampling method was utilized. This approach is effective for collecting data from large populations scattered across different geographical regions. Participants were selected from four educational institutions located in Riyadh, Jeddah, and Dhahran, representing central, western, and eastern regions of Saudi Arabia, to ensure geographic and cultural diversity. The survey was administered from January to February 2025. In the first stage, several classes were randomly selected from each university; in the second stage, students from these classes were invited to participate. Paper-based surveys were distributed to all participants, and trained research assistants aided respondents in completing the questionnaire within approximately 25 min. The assistants provided only procedural guidance (e.g., explaining instructions, collecting responses) and did not view individual answers, thereby minimizing social desirability bias. The inclusion and exclusion criteria were simplified for conciseness: only full-time students currently enrolled in university programs and aged 18–26 years were included, while part-time students and working professionals were excluded to maintain a homogeneous academic sample. In total, 700 students were enlisted; 63 (9%) were excluded due to incomplete or invalid responses on key variables. The final valid sample consisted of 627 students (376 males and 251 females). No significant demographic differences were observed between included and excluded participants. Additional details are shown in Table 1.

### Common method bias (CMB)

The use of self-administered surveys may introduce common-method bias (CMB), a measurement error arising from the research design and data-collection process. To assess potential bias, this study employed Harman’s single-factor test, in which a single factor extracted from principal component analysis should account for less than 50% of the total variance^41^. The first factor in this study explained 32.78% of the variance, which is below the threshold. However, consistent with methodological recommendations Podsakoff, MacKenzie, Lee and Podsakoff^42^; Kock, Berbekova and Assaf^43^, this test is recognized as limited, and we therefore interpret the result cautiously and suggest that future research adopt more robust methods, such as a marker variable, to further validate CMB control.

## Measurement development

### Social media addiction

The Bergen Social Media Addiction Scale (BSMAS), a six-item instrument, was used to measure SMA^44,45^. Students were asked to respond to items about their social-media use over the past year, assessing six recognized addiction dimensions: preoccupation, mood modification, tolerance, conflict, withdrawal, and relapse^23^. A sample item is: *“*Do you become restless or troubled if prohibited from using social media?” Responses were rated on a five-point Likert scale (1 = Strongly disagree to 5 = Strongly agree), with higher scores indicating stronger addictive tendencies.

### Brief family relationship scale (BFRS)

The Brief Family Relationship Scale (BFRS), a 16-item instrument developed by Fok, Allen, Henry and Team^46^, and validated by Aziz, Chemnad, Al-Harahsheh, Abdelmoneium, Baghdady and Ali^47^, was used to assess three components of family functioning: cohesion, expressiveness, and conflict. Each sub-dimension consists of three to six items measured on a 5-point Likert scale (1 = “strongly disagree” to 5 = “strongly agree”). In this study, consistent with prior research employing the BFRS in similar cultural contexts, all items were aggregated into a single composite score representing the overall family environment. This composite score was then used as the mediating variable in the structural model.

### Depression and anxiety

The Depression Anxiety Stress Scales-14 (DASS-14) was used to measure psychological distress^48^. The instrument consists of 14 items: seven assessing depression and seven assessing anxiety. The depression subscale evaluates symptoms such as dysphoria, powerlessness, and anhedonia, while the anxiety subscale captures autonomic arousal, situational anxiety, and subjective anxious affect. Higher scores indicate greater symptom severity.

### Pilot study

A pilot study was conducted before the main data collection to assess clarity, reliability, and internal consistency of the survey instruments. A sample of 67 students (10.7% of the total study population) was drawn from one participating public university and was demographically representative of the main sample. Cronbach’s α values for all subscales exceeded 0.70, confirming satisfactory reliability for use in the main study. It can be seen in Table 2.

**Table 1:** Demographic details.

Description	No.	Percentage
Gender		
Male	376	60.0
Female	251	40.0
Age		
18–21	490	78.1
22–25	137	21.9
Education (Enrolled)		
Undergraduate	498	79.4
Masters	129	20.6

**Table 2:** Building the Reliability of the Study.

Variables	CA coefficient
Social media addiction	0.874
Family environment	0.863
Depression	0.846
Anxiety	0.844

### Data analysis

AMOS 25.0 was used to test the study hypotheses using structural equation modeling^49^. We implemented the two-step structural equation modeling technique recommended by Anderson and Gerbing^50^, commencing with Confirmatory Factor Analysis (CFA) to verify model adequacy. A conclusive hypothesized model was evaluated to analyze the relationships among the variables. During the execution of confirmatory factor analysis, several fit indices were employed, including chi-square per degrees of freedom (2/df), comparative fit index (CFI), standardized root mean square residual (SRMR), Tucker-Lewis index (TLI), and root mean square error of approximation (RMSEA).

The study used the model-fit criteria suggested by Kline^51^ to interpret these indices, which indicate an appropriate model fit when CFI and TLI are ≥ 0.90 and RMSEA and SRMR are ≤ 0.08. The findings indicate that the suggested model meets the criteria for a reasonable fit (CFI = 0.91, TLI = 0.92, RMSEA = 0.05, SRMR = 0.04).

### Descriptive statistics

Table 3 reports mean, SDs, AVE, and Pearson’s correlations. All correlations among the studied variables were statistically significant. Convergent validity was supported because all AVE values exceeded 0.50. Discriminant validity was further supported by the HTMT criterion (Table 6), with all values below 0.85.

**Table 3:** Descriptive statistics.

AVE Mean SD				Correlations			
				1	2	3	4
**1. SMA**	0.64	2.12	1.33	-			
**2. FE**	0.65	1.83	1.03	0.43**	-		
**3. DEP**	0.66	1.44	0.85	0.37**	0.31**	**-**	
**4. ANX**	0.68	2.93	1.13	0.41**	0.42**	0.47**	-

AVE: average variance extracted, SMA: social media addiction, FE: family environment, DEP: depression, ANX: anxiety.

### Measurement model

This research employed “Confirmatory Factor Analysis (CFA)” Kline^51^ to evaluate the measurement model, with the loadings, alpha values, and “composite reliability (CR)” for every component provided in Table 4. The alpha values for social media addiction, home environment, depression, and anxiety among students are 0.92, 0.91, 0.90, and 0.91, in that order. These alpha values exceed the specified limit of 0.70. The factor loadings for social media addiction ranged from 0.787 to 0.853, for family environment from 0.712 to 0.845, for depression from 0.702 to 0.788, and student anxiety from 0.717 to 0.813. All factor loadings exceed 0.50 and significantly contribute. The composite reliability (CR) ranged from 0.88 to 0.912 for social media addiction, family environment, depression, and anxiety among students, exceeding the suggested threshold of 0.60^52,53^. Moreover, we performed a serial CFA to test that the model adequately represented numerous constructs. The suggested 4-factor assessment model (social media addiction, family environment, depression, and anxiety among students) demonstrated a satisfactory fit to the data: χ2 = 2693.54, Df = 945, χ2/df = 2.846, CFI = 0.91, TLI = 0.92, RMSEA = 0.05, and SRMR = 0.04 (Table 5). The suggested 4-factor measurement model is the most appropriate among the alternatives shown in Table 5. All observed items exhibited substantial loadings on their corresponding latent variables (Table 4). The suggested 4-factor model was compared with various CFA models. As shown in Table 5, all fit indices fall within recommended limits, confirming that the four-factor model provides the best representation of the data compared with alternative models. Therefore, the measurement model was considered adequate for further structural analysis.

**Table 4:** Measurement model.

Factor	Items	Loadings	S.E.	T	C.*R*.	Α
SMA	SMA1	0.792	-	-	0.91	0.92
	SMA2	0.787	0.061	12.901**		
	SMA3	0.812	0.063	12.888**		
	SMA4	0.853	0.069	12.362**		
	SMA5	0.840	0.061	13.770**		
	SMA6	0.798	0.067	11.910**		
FE	FE1	0.798	-	-	0.89	0.91
	FE2	0.715	0.062	11.532**		
	FE3	0.802	0.064	12.951**		
	FE4	0.845	0.061	12.531**		
	FE5	0.804	0.063	12.592**		
	FE6	0.794	0.065	12.761**		
	FE7	0.841	0.069	12.188**		
	FE8	0.712	0.067	10.626**		
	FE9	0.801	0.061	13.131**		
	FE10	0.806	0.067	12.029**		
	FE11	0.796	0.063	12.634**		
	FE12	0.712	0.065	10.953**		
	FE13	0.843	0.064	13.171**		
	FE14	0.803	0.061	13.163**		
	FE15	0.714	0.069	10.347**		
	FE16	0.844	0.066	12.787**		
DEP	DEP1	0.823	-	-	0.88	0.90
	DEP2	0.788	0.065	12.138**		
	DEP3	0.711	0.061	11.655**		
	DEP4	0.702	0.062	11.525**		
	DEP5	0.711	0.062	11.322**		
	DEP6	0.714	0.063	11.333**		
	DEP7	0.703	0.062	11.338**		
ANX	ANX1	0.759	-	-	0.88	0.91
	ANX2	0.811	0.062	13.080**		
	ANX3	0.762	0.063	12.095**		
	ANX4	0.764	0.061	12.524**		
	ANX5	0.813	0.064	12.703**		
	ANX6	0.717	0.065	11.031**		
	ANX7	0.806	0.065	12.400**		

**Table 5:** CFA results.

Model	χ^2^	Df	χ^2^/df	CFI	TLI	RMSEA	SRMR
4-factor model (hypothesized model)	2693.54	945	2.846	0.91	0.92	0.05	0.04
3-factor model	7731.42	958	8.062	0.77	0.80	0.13	0.12
2-factor model	7309.96	959	7.630	0.82	0.82	0.12	0.07
1-factor model	8681.33	962	9.034	0.78	0.71	0.15	0.15

**Table 6:** Discriminant validity (HTMT) criterion.

Constructs	SMA	FE	DEP	ANX
SMA	-	0.68	0.52	0.5
FE		-	0.62	0.58
DEP			-	0.62
ANX				-

The study assessed discriminant validity using the heterotrait-monotrait ratio (HTMT^54^. HTMT offers a more reliable evaluation than traditional methods, such as the Fornell-Larcker criterion, by comparing between-construct and within-construct correlations. The HTMT value in Table 6 between the constructs SMA and FE is 0.68, indicating that, despite their association, they are empirically different. Likewise, constructs such as FE and DEP demonstrate an HTMT value of 0.62, thereby strengthening their discriminant validity. All HTMT values in the analysis were below the rigorous threshold of 0.85, confirming that the constructs are both empirically and conceptually distinct^55,56^. This validates the measurement model and improves the structural analysis by mitigating multicollinearity or construct overlap.

### Hypothesis testing

We employed structural equation modeling (SEM) with maximum likelihood estimation using AMOS to test the proposed research hypotheses. Consistent with the correlations reported in Table 3, the SEM results presented in Table 7 provided empirical support for H1 and H2. Specifically, social media addiction (SMA) showed a significant positive association with depression (β = 0.431, t = 7.836, *p* < 0.01), supporting H1. Likewise, SMA was positively associated with anxiety (β = 0.312, t = 5.114, *p* < 0.01), thereby supporting H2. To examine the mediating role of the family environment (H3 and H4), a bootstrapping mediation analysis with 5,000 resamples and 95% bias-corrected confidence intervals was conducted (Table 8). For H3, the direct effect of SMA on depression became non-significant after including the family environment (β = 0.043, S.E. = 0.060, t = 0.716, 95% CI [− 0.074, 0.160]), whereas the indirect effect through family environment was significant (β = 0.148, S.E. = 0.062, t = 2.387, 95% CI [0.027, 0.269]). These results indicate that the family environment fully mediates the relationship between social media addiction and depression, thus supporting H3. Similarly, for H4, the indirect effect of SMA on anxiety via family environment was statistically significant (β = 0.162, S.E. = 0.062, t = 2.612, 95% CI [0.041, 0.283]), while the direct effect of SMA on anxiety became non-significant (β = 0.007, S.E. = 0.060, t = 0.116, 95% CI [− 0.111, 0.125]). This pattern confirms the full mediating role of the family environment in the relationship between social media addiction and anxiety, supporting H4. Overall, H1 and H2 were supported in the direct-effects model (Table 7), whereas H3 and H4 were supported in the bootstrapped mediation model (Table 8), demonstrating full mediation. Following AMOS reporting conventions, significance levels are indicated as *p* < 0.001, *p* < 0.01, and *p < 0.05. All direct paths reported in Table 7 were statistically significant at *p* < 0.001. Consistent with established mediation criteria, indirect effects were considered significant when zero was not included in the confidence interval^57^.

**Fig. 2:**
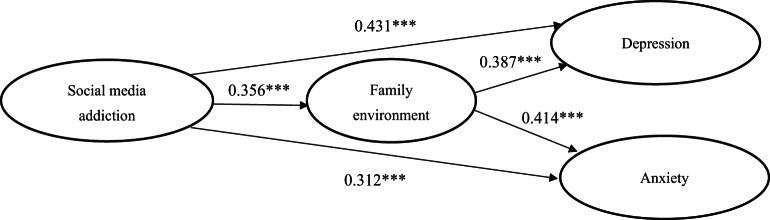
Final Structural Equation Model Results.

Figure 2 presents the final structural equation model with standardized path coefficients among social media addiction, family environment, depression, and anxiety. The results show that social media addiction is significantly associated with the family environment, which in turn has significant relationships with both depression and anxiety. Consistent with the bootstrapping results (Table 8), the direct effects of social media addiction on depression and anxiety become non-significant after accounting for the family environment, indicating full mediation. Overall fit indices indicate excellent model fit (χ²/df = 2.85, CFI = 0.91, TLI = 0.92, RMSEA = 0.05, SRMR = 0.04).

**Table 7:** Direct effect.

Relationship	Β	S.E.	T	95% CI
SMA ➜ DEP	0.431	0.055	7.836	(0.382, 0.512)
SMA ➜ ANX	0.312	0.061	5.114	(0.209, 0.471)
SMA ➜ FE	0.356	0.071	5.014	(0.311, 0.602)
FE ➜ DEP	0.387	0.057	6.789	(0.276, 0.498)
FE ➜ ANX	0.414	0.062	6.677	(0.397, 0.585)

Note: All direct paths reported in Table 7 were statistically significant at *p* < 0.001.

**Table 8:** Bootstrapping indirect effects.

Relationship	β	S.E.	t	95% (CI)	
				Lower Limit	Upper Limit
SMA ➜ DEP	0.043	0.060	0.716	−0.074	0.160
SMA ➜ ANX	0.007	0.060	0.116	−0.111	0.125
SMA ➜ FE ➜ DEP	0.148	0.062	2.387	0.027	0.269
SMA ➜ FE ➜ ANX	0.162	0.062	2.612	0.041	0.283

## Discussion

The present study examined how the family environment mediates the relationship between social media addiction (SMA) and psychological outcomes—depression and anxiety—among university students in Saudi Arabia. SMA was positively associated with both depression and anxiety, while a supportive family environment fully mediated these links. Although the results are cross-sectional, they are consistent with prior evidence showing that excessive social media use correlates with poor mental-health outcomes among young adults^58^. Unlike previous work primarily based in Western settings, this study demonstrates that family dynamics in a Saudi, non-WEIRD context play a central role in shaping students’ emotional responses to online engagement. In a collectivist society characterized by strong parental authority and interdependence, cohesive and communicative families appeared to buffer the negative psychological impact of social media by fostering positive coping strategies, enhancing self-esteem, encouraging emotional regulation, and guiding balanced media use through parental monitoring and open dialogue. These processes reduce the emotional vulnerability that amplifies anxiety and depression symptoms. These findings extend existing SMA frameworks by embedding social-comparison and mood-enhancement theories within a collectivist cultural model that better reflects Middle-Eastern realities^21,39,59,60^. The key contribution of this study lies in highlighting the cultural mechanisms through which family cohesion mitigates the mental-health risks of SMA. In Saudi Arabia—one of the most digitally connected nations globally—familial bonds remain a crucial social resource. Strengthening these ties can therefore serve as a culturally congruent strategy for promoting students’ digital well-being. From a policy perspective, universities could collaborate with families to deliver culturally grounded digital-literacy and well-being programs, including joint workshops, parent–student awareness sessions, and peer initiatives that encourage responsible online behavior. Such interventions would complement individual counseling efforts by leveraging family influence as a protective factor. Finally, while the present study advances understanding of SMA in a high-connectivity yet collectivist context, its cross-sectional design limits causal inference. Future longitudinal or experimental research should examine the temporal direction of these associations and test moderating effects such as gender, socioeconomic status, and regional variation.

### Implications

The research possesses both theoretical and practical ramifications. This study theoretically enhances the literature by illustrating the mediatory role of family environment in the association between SMA and both depression and anxiety among university students. The findings underscore the need to design youth-focused therapies that integrate digital-well-being components—such as cognitive-behavioral training for emotion regulation and workshops that promote mindful online engagement. The study also highlights the shared duty of parents in combating youngsters’ social media addiction. Family-based interventions could include parental guidance programs that teach communication strategies, early detection of problematic online behavior, and collaborative rule-setting for screen-time management. Evidence-based preventative and intervention techniques must take into account the impact of family environments. Involving parents in effective preventative methods is a critical element in reducing the hazards associated with social media addiction. Policymakers and educational institutions could collaborate to implement school-family partnerships that cultivate supportive digital environments for adolescents.

### Limitations and future research prospects

This study has several limitations that should be acknowledged. First, the sample was drawn from four public educational institutions in the Kingdom of Saudi Arabia, which may limit the generalizability of the results. Future research could include private universities and institutions from additional regions of the Kingdom to provide a more representative picture of the student population. Second, the study relied on self-reported measures of social media use, depression, anxiety, and family environment. Such self-reports may introduce recall or social-desirability bias, potentially affecting the precision of responses. Future work should incorporate multi-informant data, such as parent or peer reports, or use objective behavioral metrics (e.g., digital-use logs) to strengthen data accuracy. Third, the study used assessments of depressive and anxiety symptoms rather than formal diagnostic tools; therefore, the findings cannot be generalized to clinical populations. Future studies should examine these associations within clinical or high-risk groups to validate the observed relationships. Fourth, the cross-sectional design restricts causal inference. While the present results indicate statistically significant associations and mediation effects, these should be interpreted as correlational rather than causal. Future research employing longitudinal or experimental designs is necessary to confirm the directionality of the impact and to clarify the temporal sequence among social media addiction, family environment, and mental-health outcomes. Finally, although Harman’s single-factor test was used to assess common-method bias, this approach has well-recognized limitations and may not fully capture all sources of bias. Future studies could employ more robust methods, such as the marker-variable technique or latent method factor approaches, to ensure more rigorous validation of measurement models. Despite these limitations, the study offers novel insights by highlighting the mediating role of the family environment in a Saudi/non-WEIRD context. These findings provide a solid foundation for future cross-cultural and intervention research addressing the psychological risks of excessive social-media use among university students.

## Conclusion

In conclusion, this study demonstrates that the family environment fully mediates the relationship between social media addiction and psychological distress, specifically depression and anxiety among Saudi university students. Supportive and communicative family relationships substantially buffer the emotional harm of excessive social media use, underscoring the protective value of strong familial bonds. From a policy and practice standpoint, Saudi universities should integrate culturally responsive digital well-being programs and mental health counseling that address both online behavior and family interactions. Policymakers and educational authorities can design joint awareness initiatives promoting balanced media use, parental involvement, and healthy communication patterns. Families, in turn, can reinforce these efforts by setting shared technology boundaries and encouraging more offline social engagement. Future interventions should remain culturally grounded, reflecting the collectivist family structures that shape coping strategies and emotional resilience among young people in Saudi Arabia.

## Data Availability

The datasets produced and/or analyzed in this study are not publicly accessible; however, they can be obtained from the corresponding author upon request.

## Declarations

All procedures complied with institutional and international ethical standards. Written informed consent was obtained from all participants prior to data collection, and participants were assured of confidentiality and anonymity.
